# Management of recurrent haemoptysis in malignancy with combined TISSEEL and intrabronchial valves

**DOI:** 10.1002/rcr2.406

**Published:** 2019-01-31

**Authors:** James Di Michiel, Corinna Pan, Alvin Ing, Tajalli Saghaie

**Affiliations:** ^1^ Department of Thoracic medicine Concord Repatriation General Hospital Sydney New South Wales Australia; ^2^ Faculty of Medicine and Health Sciences Macquarie University Sydney New South Wales Australia

**Keywords:** Bronchoscopy, endobronchial valves, fibrin sealant, haemoptysis, malignancy

## Abstract

Management of recurrent haemoptysis poses a difficult clinical scenario. TISSEEL (Baxter Healthcare Corporation Deerfield, IL, USA) is a fibrin sealant often used in surgery to aid control of bleeding. However, when endoscopic TISSEEL is used independently, migration, dislodgement, or even expectoration of the fibrin clot is a common complication that may lead to recurrence of haemoptysis. Here we describe two patients with recurrent haemoptysis in the context of malignancy who underwent bronchoscopy, during which they were managed with application of topical TISSEEL to the bleeding area, followed by deployment of an intrabronchial valve, followed by a further application of TISSEEL over the valve. The combination of TISSEEL and intrabronchial valve appeared to control haemoptysis and was resistant to expectoration or migration in these two cases. Thus, this combination may provide a safe and effective therapeutic option for the control of bronchial bleeding secondary to malignancy.

## Introduction

Management of recurrent haemoptysis poses a difficult clinical scenario. Current standard management options for localized endobronchial bleeding include bronchoscopy with instillation of topical iced saline or vasoconstrictive agents such as adrenaline, endobronchial laser therapy, argon plasma coagulation therapy, bronchial artery embolization or surgery. TISSEEL (Baxter Healthcare Corporation, Deerfield, IL, USA) is a fibrin sealant often used in surgery to aid control of bleeding. There are case reports describing the effectiveness of fibrin sealants such as TISSEEL in recurrent haemoptysis when other techniques have failed or are not indicated 1, 2, 3, 4. However, when endoscopic TISSEEL is used independently, migration, dislodgement, or even expectoration of the fibrin clot is a common complication that may lead to recurrence of haemoptysis 5, 6. We describe successful management of recurrent haemoptysis in the context of malignancy in two cases using combined intrabronchial valves (IBVs) and the fibrin sealant TISSEEL.

## Case Reports

### Patient 1

**Figure 1:**
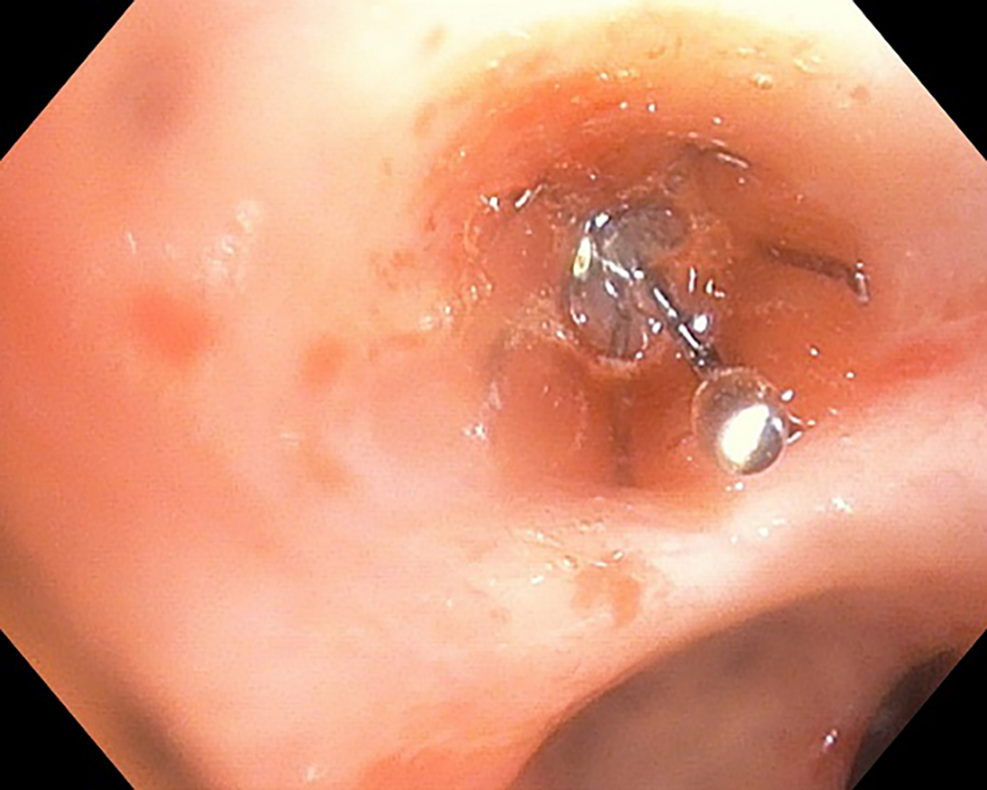
Deployment of intrabronchial valve in right middle lobe following initial application of TISSEEL.

**Figure 2:**
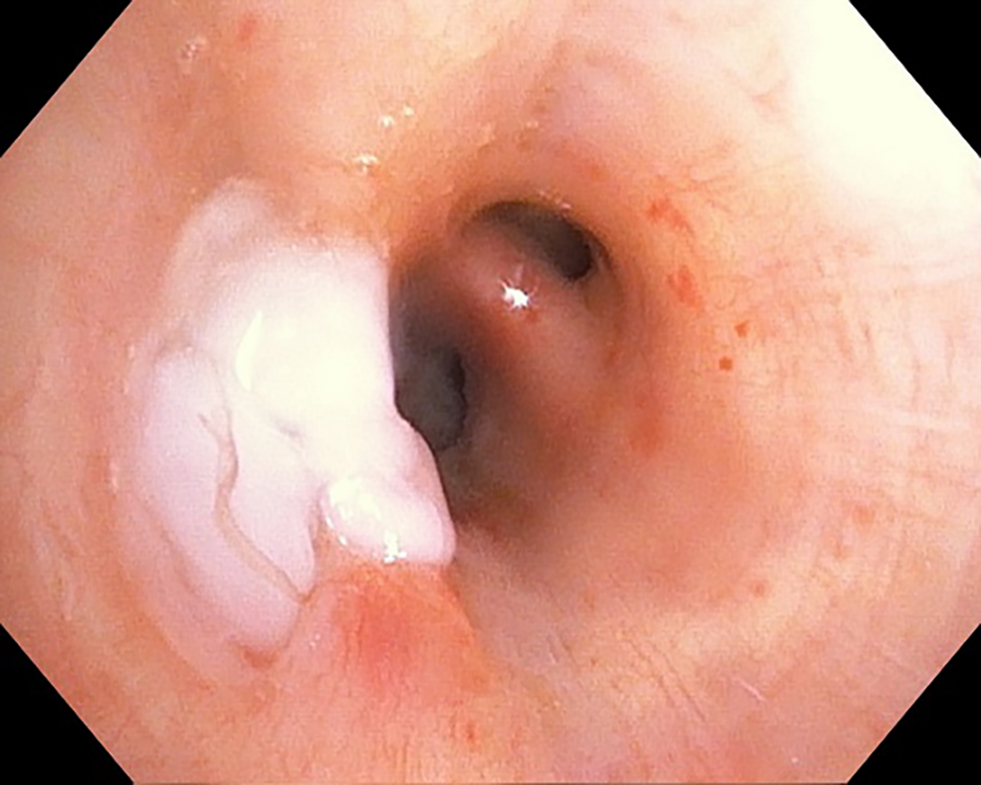
Second application of TISSEEL placed over intrabronchial valve in right middle lobe.

A 44‐year‐old woman with a history of metastatic triple negative breast cancer and lung metastases presented with a six‐month history of recurrent haemoptysis. She had no other significant medical history. She was initially managed for her right breast cancer with a wide local incision and adjuvant chemoradiotherapy in 2014; however, her malignancy recurred two years later. She had positive margins on subsequent right mastectomy and proceeded to excision of the right pectoralis major and overlying dermis. Six months later she was found to have bilateral pulmonary metastases and underwent initial diagnostic bronchoscopy identifying a bleeding mass in the medial segment of the right middle lobe (RB5), which was subsequently treated with topical adrenaline and biopsied – confirming metastatic disease. Her malignancy progressed despite palliative chemotherapy with epirubicin and cyclophosphamide, during which time she developed worsening haemoptysis of ~1/2 cup (~120 mL) daily. A multidisciplinary decision was then made to perform therapeutic bronchoscopy due to excessive distress caused to the patient because of haemoptysis. She underwent bronchoscopy using a therapeutic video bronchoscope (Olympus BF‐TH190, Olympus Corporation, Tokyo, Japan) introduced via a rigid bronchoscope, which provided secure airway access. Endobronchial survey revealed the source of bleeding in the distal right middle lobe, although the actual bleeding source was not directly visible. A volume of 2 mL of TISSEEL was injected into the right middle lobe bronchus via a catheter followed by deployment of a size 6 Spiration (Redmond, WA, USA) IBV to add stability and prevent expectoration (Fig. 1). A further 1 mL of TISSEEL was then applied over the valve (Fig. 2). The procedure abolished the patient's haemoptysis instantly.

Two weeks later the patient developed recurrent haemoptysis; however, repeat bronchoscopy showed a different source of bleeding in the right lower lobe, with the existing combination TISSEEL and IBV still in place in right middle lobe and maintaining haemostasis. To control the new bleeding TISSEEL was injected in the right lower lobe bronchus distal to the opening of RB6, followed by deployment of a size 9 IBV. Further injection of TISSEEL was then applied and haemostasis was achieved. Unfortunately the patient was found to have brain metastases and died of her malignancy 10 weeks later, without recurrence of haemoptysis.

### Patient 2

A 67‐year‐old woman with a history of papillary thyroid carcinoma and known bilateral lung metastases presented with several weeks of recurrent haemoptysis. Her initial thyroid carcinoma was managed with thyroidectomy and neck dissection in 1998; however, it recurred with lung metastases initially found in 2007 and managed conservatively. Her other comorbidities included grade 3 ductal carcinoma in situ managed with radiotherapy, type 2 diabetes mellitus, hypertension, gastro‐oesophageal reflux disease, and osteoarthritis. She was not on any anti‐platelet or anti‐coagulant medication. Her volume of haemoptysis was mild with <100 mL daily. She underwent therapeutic bronchoscopy using a video bronchoscope (Olympus BF‐T180) introduced via a rigid bronchoscope. The source of bleeding was identified to be originating from the lateral basal segment of the left lower lobe (LB9). A volume of 1 mL of TISSEEL was then injected into distal LB9, followed by deployment of a size 6 Spiration IBV, followed by a further 1 mL of TISSEEL to cover the valve. There were no complications during the procedure and patient's haemoptysis resolved. Eight months later she developed recurrent haemoptysis with repeat bronchoscopy showing bleeding originating from the posterior basal segment (LB10). The previous IBV was found to be in place in LB9. A volume of 2 mL of TISSEEL was injected in LB10, followed by deployment of a size 7 IBV and a further 2 mL of TISSEEL over the LB10 valve. There was excellent seal after the procedure with resolution of haemoptysis.

## Discussion

TISSEEL (combination of synthetic aprotinin, factor VIII, and fibrinogen combined with thrombin; Baxter Healthcare Corporation) is a fibrin sealant indicated for use as an adjunct in patients undergoing surgery when control of bleeding by conventional surgical techniques is ineffective or impractical. Although fibrin sealants are widely used in general surgical procedures, there is limited data describing their use in control of haemoptysis 1, 2, 3, 4. In the largest study, Tsukamoto et al. showed that combination fibrinogen–thrombin was effective in long‐term control of haemoptysis in 11 (79%) out of 14 cases 3. de Gracia et al. looked at patients with severe haemoptysis (≥150 mL in 12 h) who received endobronchial topical fibrin–thrombin treatment, with control of bleeding seen in seven (70%) out of 10 patients 4. However, the existing literature shows that migration, dislodgement, and even expectoration of the fibrin clot is not uncommon and often requires repeat procedures 7, 8. While case reports of the use of IBVs for recurrent haemoptysis [9] have been described, there is no previous literature involving combination fibrin sealants such as TISSEEL and IBVs for managing this condition.

It is hypothesized that IBVs would stabilize the fibrin sealant and prevent expectoration or migration. Here we describe the use of combination TISSEEL and IBVs for the management of recurrent haemoptysis secondary to malignancy. This combination therapy, to the best of our knowledge, represents a novel intervention for palliative management of this condition. Our two patients both had immediate resolution of their symptoms without significant adverse events, which suggests that combination TISSEEL and IBV therapy may be a safe and therapeutic option for the control of bronchial bleeding secondary to malignancy.

### Disclosure Statement

Appropriate written informed consent was obtained for publication of these case reports and accompanying images.
